# Keeping it under lock and keywords: exploring new ways to open up the web archives with notebooks

**DOI:** 10.1007/s10502-022-09391-6

**Published:** 2022-07-04

**Authors:** Leontien Talboom, Mark Bell

**Affiliations:** 1 University College London, London, UK; 2 The National Archives, Richmond, UK

**Keywords:** Notebooks, Digital archiving, Web archives, Access, Finding aids, Computational archival science

## Abstract

The UK Government Web Archive (UKGWA) has been archiving government websites since 1996 and now holds regular snapshots of over 5000 sites. Currently, this material can be accessed through browsing or a simple keyword search interface on their website and has also been catalogued in The National Archives’ online catalogue, Discovery. However, the scale of the UKGWA exposes the limits of the current search interface, and there is no facility to understand the archive in aggregate. This article seeks to go beyond the simple keyword search by exploring the data sources available, from APIs to web crawling, for computational analysis of the UKGWA. The article is accompanied by two Python Notebooks which present examples of analysis using each data source. Notebooks lower the technical barriers for the reader to explore and interpret the UKGWA as data, while surfacing the challenges around making web material computationally accessible.

## Introduction

In recent years, archives and other memory institutions have collected increasing volumes of born-digital material. This new material has not only disrupted the way that memory institutions acquire and preserve this material, but also how this material is accessed. This is reflected in new areas of study such as ‘digital humanities’ and ‘computational archival science’ (Goudarouli 2018), the emergence of networks specifically dedicated to digital material (Netwerk Digitaal Erfgoed 2019; AURA Network 2021), and by the digital strategies published by memory institutions and forthcoming projects for their collections and materials (The British Library 2017; The National Archives 2017).

In line with the recent ‘Data as Collections’ (Padilla et al. 2018) project, this article aims to explore the UK Government Web Archive (UKGWA) by the use of computational methods. The UKGWA has been selected for this work as it is one of few large born-digital collections currently available without any major restrictions. UKGWA has been already used as an example dataset for workshops and study groups (Storrar and Talboom 2019; Beavan et al. 2021), although these used subsets of the collection and no exploration of the collection in full has been conducted. To provide demonstrations of the article’s discussion points, the authors have created two Python Notebooks for the reader to view or interact with. The Notebooks showcase available data and possibilities beyond keyword searching, and to highlight some of the issues encountered when accessing UKGWA (The National Archives 2021k) computationally. They are aids to discussion rather than demonstrations of novel computational techniques and can be accessed by following the links from the GitHub repository (Bell 2021).

After introducing these case studies, the article will discuss the drawbacks and possibilities of this new approach to archival search for web archives and the feasibility of using Notebooks with regard to skills and resources needed to implement and use them.

## Accessing web archives as data

The born-digital material that is the focus of this article is archived web material. In the last two decades, the use of the web has increased exponentially, leading to a huge amount of important information being found online. Rapidly evolving technologies and the relatively short life spans of many websites has led to memory institutions urgently archiving web materials with an emphasis on capturing them before they disappear ahead of integration with their more traditional collections. This web material is dynamic and diverse, ranging from published scholarly articles to government campaigns (International Internet Preservation Consortium 2021b).

Within the research community, there is a growing interest in archival web material. The Internet Archive was founded in 1996 and UKGWA holds archived websites from the same year, making web archives a primary source for historians of the late twentieth and early twenty-first centuries. A number of events have focused specifically on web archives including the Web Archiving Week (International Internet Preservation Consortium 2017), hosted by the British Library and the School of Advanced Study, University of London, The Web Archiving Conference (International Internet Preservation Consortium 2021a), The Research Infrastructure for the Study of Archived Web material (RESAW) conferences (RESAW 2021), and the Engaging with Web Archives Conference (Engaging with Web Archives 2021).

When looking at the programmes in detail, most of the focus is on fostering collaborations, discussing preservation and capture strategies and talking through individual research projects that have used archival web material. Only a small number of talks are on the access to this material and the computational methods that may enhance the access to it.

When looking at it in practice, a growing number of institutions are preserving this material and therefore the amount of archived web material accessible online is also growing. Not all of this material can be accessed in the online environment, mainly due to certain (predominantly copyright) restrictions. For example, both the British Library and the Dutch National Library only provide access to parts of their collections from their reading rooms (British Library 2021b; Koninklijke Bibliotheek (National Library of the Netherlands) 2021). Open material is generally accessed through a keyword search interface or an online catalogue; examples include the Wayback Machine of the Internet Archive (Internet Archive 2021a) and the search interfaces of the UK Web Archive (UK Web Archive 2021) and UKGWA (The National Archives 2021e). These online catalogues and their simple keyword search systems have received a growing number of critiques in the last decade.

First off, users are not used to navigating online catalogues. A survey conducted in the Netherlands on how academics search for digital material, summed it all up with the phrase ‘Just Google It’, the prevalent digital search method. Google was used to find documents within a memory institution, as the online catalogue was found to be too difficult to navigate (Kemman et al. 2012). This is not surprising, as Gollins and Bayne (2015) point out, the original catalogues held by archives, which have now been made into online versions, were made for archivists by archivists. A catalogue is difficult to navigate by a user who is more familiar with a Google or Wikipedia search system (Gollins and Bayne 2015, 131–33). Memory institutions are aware of this problem and know that a large number of users come in through Google. The Europeana portal indexed their material and saw a 400 per cent increase of pages visited (Nicholas and Clark 2015, 28).

Secondly, the simple keyword search, the most popular approach for online catalogues and web archive interfaces, has received a lot of criticism. As Gilliland (2016) and Winters and Prescott (2019) point out, keyword search may not be enough to actively engage with the digital archival material (Gilliland 2016; Winters and Prescott 2019). Also Putnam (2016) and Romein et al. (2020) warn about the pitfalls of using search engines, as they are not transparent and can be biased, influencing the research that is conducted (Putnam 2016; Romein et al. 2020). Furthermore, users themselves are changing and seem to be wanting other search options that go beyond keywords. Milligan (2019) examined the research methods of historians, and he discovered that querying databases and parsing data, to just name a few examples, are slowly becoming more common research methods for historians (Milligan 2019).

Thirdly, the material itself is very different to traditional archival collections. With the sheer volume of digital material, and specifically born-digital material, it is impossible to index everything to improve searchability. There have been successful attempts to automate part of this process (Corrado 2019; Saleh 2018), but there is also a movement of ideas towards the benefits of this material being digital. This shift in thinking started around the 2010s where the digitised material went from being classed as digital surrogates to being seen as enriched and useful data in itself (Nicholson 2013). These collections can be processed by computers garnering new insights from the material and the number of projects focusing and advocating for seeing this material as data is growing, including ‘Collections as Data’ (Padilla et al. 2018), ‘Digging Into Data’ (Digging into Data Challenge and Trans-Atlantic Platform 2019), and ‘Plugged in and Powered Up’ (The National Archives 2019).

Projects within the web archiving community have also actively explored ideas beyond keyword search. The Web Archive Retrieval Tools (WebART) ran from 2012 to 2016 and aimed to critically assess the value of web archives, and developed a number of tools to access web material. The focus of the projects was on realistic research scenarios that would be conducted by humanities researchers. The project produced a number of papers and conferences and a tool named WebARTist, a Web Archive access system, but it is sadly, and perhaps ironically, no longer accessible (WebART 2016).

The Alexandria project also developed tools aimed at developers to make it easier to explore and analyse web archives. Their focus was the semantics of the web archive, looking at ways of exploring web archival material without indexing it. They developed an entity-based search called ArchiveSearch which makes it possible to use most concepts of Wikipedia as search terms (Kanhabua et al. 2016).

As part of the Big UK Domain Data for Arts and Humanities (BUDDAH), the SHINE tool, a prototype historical search engine, incorporates a trend analysis option alongside a more traditional search function (British Library 2021a). Archives Unleashed uses the tools of Big Data to enable large-scale analysis and has teamed up with Archive-IT to become more sustainable. Their experimental tools have surfaced in multiple ways (Ruest et al. 2020), notably through Notebooks which are growing in popularity when showcasing tools online. GLAM Workbench has now also teamed up with a number of Web Archives and has introduced a set of Notebooks aimed at exploring Web Archives (Sherratt 2020).

A slightly different approach has been taken by the Dark and Stormy Archives project, which investigated the use of storytelling as a way to explore web archives. The data came from the expansive Archive-It collection from Internet Archive (Jones et al. 2020).

The UKGWA themselves have actively engaged in research going beyond the keyword search. During a Computational Archival Science (CAS) Workshop, the tools of Network Analysis were experimented with (Storrar and Talboom 2019), and an Alan Turing Institute Data Study Group explored the possibility of searching the web archive for computationally generated trends and topics (Beavan et al. 2021).

The use of Notebooks follows the trend set by the GLAM Workbench (Sherratt 2021) and The National Library of Scotland, who have released a set of Notebooks exploring their collections (National Library of Scotland 2020). Notebooks are an ideal environment to experiment with different techniques, which is easily accessible and sustainable enough for the time being (Candela et al. 2020). Also, they offer the possibility to explore an interface that goes beyond the search box. Whitelaw (2015) argues that this is the way forward and names this approach ‘generous interfaces’ (Whitelaw 2015). The CLARIAH project gives an example of using Jupyter Notebooks to showcase a generous interface similar to Whitelaw’s proposal (Wigham et al. 2019). The computational methods discussed in the case studies are not novel, but rather taken from popular open-source libraries. The aim is to combine these technologies with the available data source within the archive to showcase possibilities for exploring it as data.

## Exploring the UK Government Web Archive

The UK Government Web Archive (UKGWA) captures and preserves information that has been published by UK government institutions on the web since 1996. This includes not only websites, but also social media services such as Twitter and Youtube (The National Archives 2021c). Currently, this material can be accessed by using the search function on the UKGWA website (The National Archives 2021k). This is a standard word and phrase search, supporting boolean syntax, with the additional options of searching within a single website, and filtering by year, and resource format. The UKGWA is also catalogued within the main archive, and through an A-to-Z list providing an overview of all archived websites on the UKGWA’s own website (The National Archives 2021a).

**Fig. 1:**
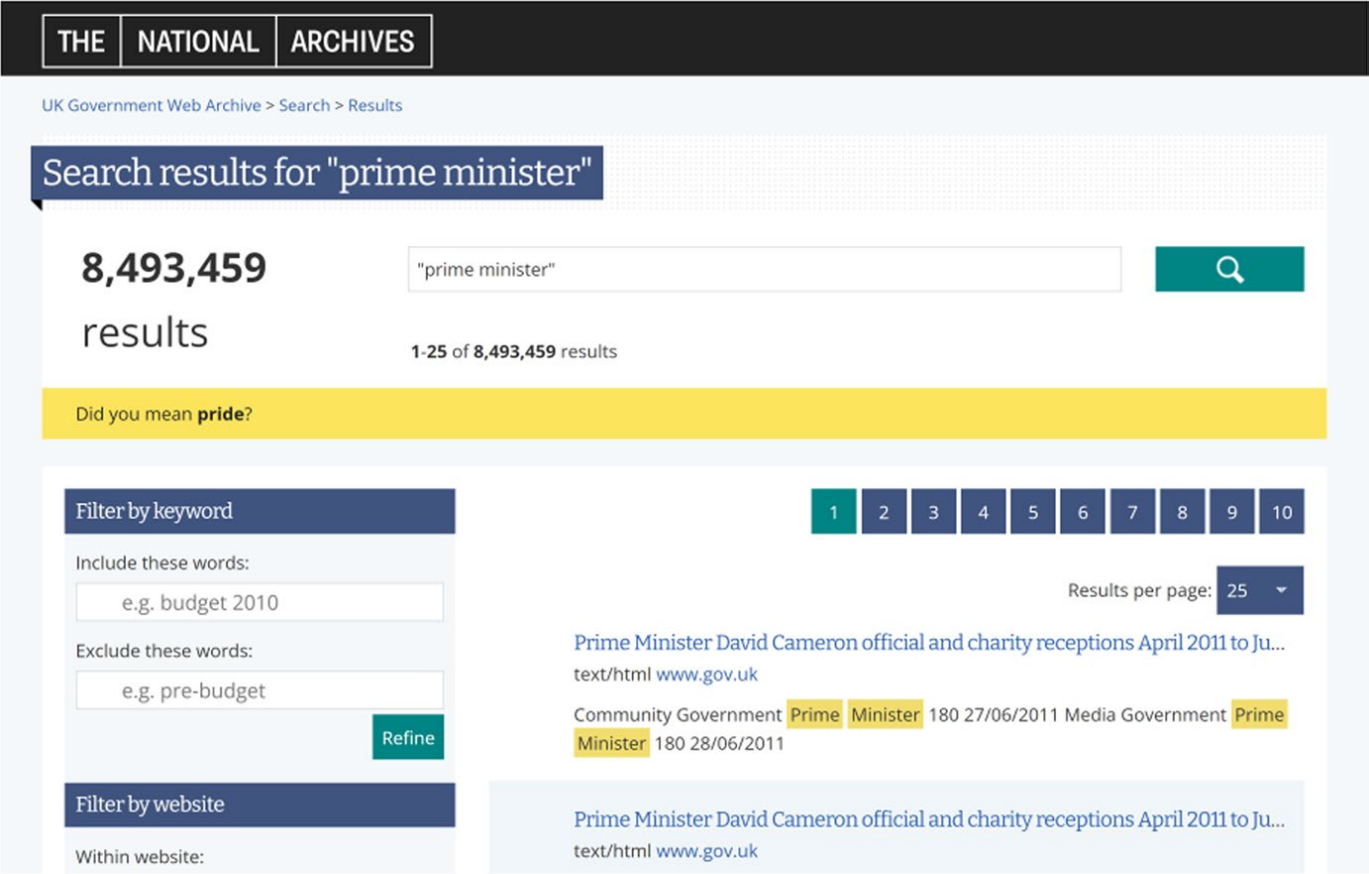
Screenshot of the search result for ‘prime minister’ in the UKGWA

Websites within the UKGWA are captured multiple times throughout a year, and crawls are performed deeply to ensure good coverage of all of the pages and resources (e.g. pdf files) within a site. To illustrate the sheer amount of these data, Fig. 1 shows an example of a search (on 30 March 2022) conducted for the phrase ‘prime minister’. As can be seen, 8,493,459 results are returned (figures in the article are correct at time of writing but are likely to change). It is possible to refine these results with the help of filters on the left hand side of the page, but their effect is limited. 96.5% of the results are HTML so filtering by type (choices: HTML, PDF, CSV, Text, Word) is only helpful if they are to be excluded. Filtering by year is most effective pre-2003, but otherwise each year yields  tens to hundreds of thousands of results, peaking at 2.6 million for 2020. It is also possible to search within a single domain, or to exclude one domain, but it is not possible to include/exclude multiple domains. There is also no ordering by relevance as might be expected with a commercial search engine, and multiple snapshots of a page will appear in the results if anything has changed on that page. Only the first 5000 results are accessible, so a user willing to look through a large number of results will still be limited by the amount the interface is able to generate for that specific search, and there is no guarantee they will see all of the pages matching their search query.

An alternative route to exploring the web archive is through TNA’s online catalogue, Discovery. There is no current functionality for searching directly for UKGWA sites, but searching for ‘web’ AND ‘snapshots’ at the series level is a reliable method for finding archived websites and returns 5032 results (The National Archives 2021f). For most of these websites, Discovery provides additional information to that found in the UKGWA A–Z list. The site is placed in the context of a department although it should be noted that websites are held at the level of a division and there is no alignment of sections of the site with non-web documents from the same department. Many entries include an administrative history (see for example https://discovery.nationalarchives.gov.uk/details/r/C16989) which provides contextual background. A link is provided from the Discovery record to the Archive Timeline page which lists the available snapshots for the site in the web archive (see https://webarchive.nationalarchives.gov.uk/ukgwa/*/http://www.vpc.gov.uk/).

## The Notebooks

Two Notebooks accompany this article, both hosted in the GitHub repository https://github.com/nationalarchives/UKGWA-computational-access, along with associated library code and data. The GitHub page includes links to open the Notebooks on three platforms: NBViewer, Google Colabs (Google 2020), and MyBinder (The Binder Team 2017). Which to select will depend on personal preference, but generally NBViewer will suit the non-technical user who wishes only to read the Notebook and view data and visualisations. Both Colabs and MyBinder are browser-based computational environments which allow the user to execute the code within the Notebook, either to refresh the data, try different parameter options, or even write their own methods to interact with the data, depending on their level of technical sophistication. Interacting with the code should not put off the non-technical user as there are directions for how, and when, to edit parameters, and there are also occasions when values can be selected from a dropdown list. The code is kept as simple as possible with just enough shown to support the narrative of the Notebook, while more complex functions are available to view in the GitHub repository for those who are interested.

The next sections describe the Notebooks and explain certain concepts in more detail. It is recommended to read this section alongside the accompanying Notebooks. A wider discussion on the use of Notebooks will follow after this section.

### Notebook 1: Data sources for exploring the UKGWA

The TNA Catalogue, Discovery, can also be accessed through an application programming interface (API). Using an API a user sends instructions to a server and database maintained by an institution. These instructions, commonly called a request, are then processed, and data are returned to the user (Hoffman 2018). It is a more convenient way of extracting data for computational analysis than downloading through the online search interface. With knowledge of the series a website is held in, the API can be used to directly navigate from a starting point in the catalogue hierarchy to the record for the website (the starting point could be the record itself with the full catalogue reference to hand). Having retrieved the record one can extract the description, administrative history (if available), and standard catalogue metadata such as parent department, series, covering dates, and taxonomy categories (Underdown 2018; The National Archives 2021b).

A more likely scenario for an API user is to extract the records of all websites, or those for a particular subject area. The same result achieved through the website search can be replicated, but accessing data directly removes the need for manual steps such as updating data when changes have been made to the catalogue.

The UKGWA’s online interface provides two routes to discovery: browsing an A–Z list of sites (The National Archives 2021a) and keyword search. To access either of these sources computationally, web crawling needs to be resorted to. The A–Z list is the easier option and is a good starting point for a researcher who wishes to understand the scope of the archive. With a little knowledge of the underlying structure of the page, it is relatively easy to extract a list of URLs and descriptions of all websites.

**Fig. 2:**
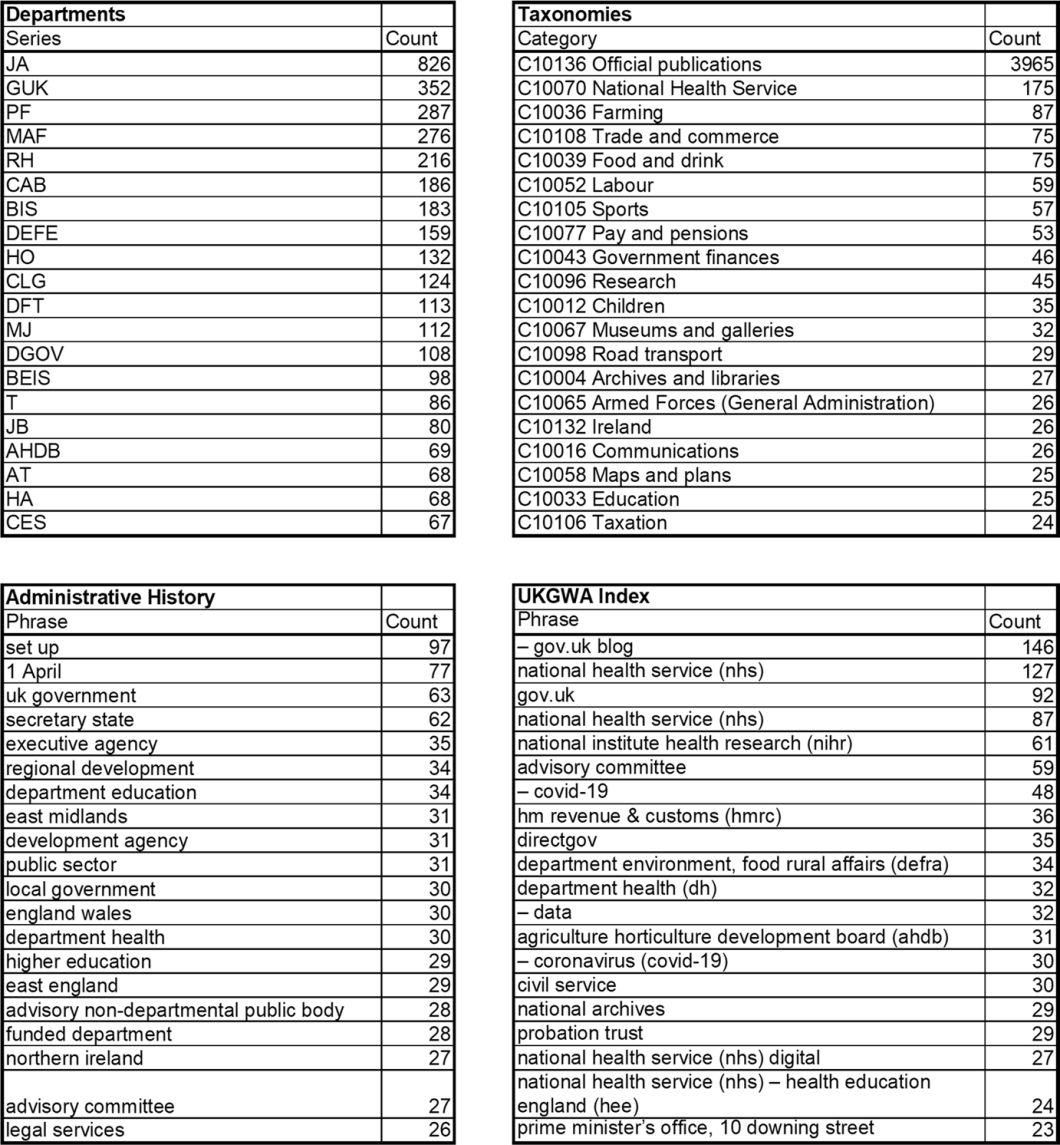
Summaries of Web Archive records by Department, Taxonomy Category, Administrative History common phrases, A–Z common phrases

The ‘Computational Access to UKGWA’ Notebook generates summary counts of records by several metadata fields: department code, start date, taxonomy category, and common phrases extracted from the description and administrative history fields. Figure 2 shows the top 20 values for four of these fields. Each table gives a different view of government on the web. The taxonomies list provides a functional view of the archive and includes typical language of government (‘executive agency’, ‘development agency’, ‘funded department’). The UKGWA index contains a mixture of department names, and some overlapping terms with the administrative history (‘advisory committee’). There is also a contrast between the curated categorisations of the archival catalogue (department codes, taxonomy categories), and programmatically extracted phrases, which include phrases like ‘set up’, dates, and multiple entries for the same subject (COVID-19).

There is a summary of common phrases from the catalogue description, but with top results such as “series contains dated gathered versions (or 'snapshots')” it is clearly not of use for understanding the archive in aggregate. There is also an obvious difference in the counts between tables, even when the text subjects appear in alignment. The GUK department (352) has more records than UKGWA’s gov.uk blog (146) and gov.uk (92) entries combined (238) because not every site listed with GUK is explicitly identified as gov.uk in the A–Z index. The taxonomy categories have a different purpose, defining records by subject matter rather than department or internet domain—almost 4000 sites are defined as ‘Official publications’. The Department of Health (JA) and the National Health Service feature at, or near, the top of three of the tables, but ‘department health’ is much lower down in the administrative history table. Other topics such as Children, East Midlands, and COVID-19 only appear once. The latter’s prevalence in the UKGWA list a result of the Archive’s effort to capture, and make available, the government’s responses to the pandemic.

Discovery includes two dates—record start and end—which can be summarised, but the end date is less informative, mostly defaulting to 2100. Dates are extracted from UKGWA using a public facing API known as CDX (Alam et al. 2016), an index format created by the Internet Archive’s open-source Heritrix web crawler (Internet Archive 2021b). The CDX API can be used to extract a full archiving history of a url captured in the archive. A request to the API for a url returns one row for each capture of the page, in various formats. There are 11 fields per row, of which the Notebook uses three: timestamp, status code, and digest. The timestamp is used to analyse when and how often a page has been archived. While it is possible through the search interface to filter by date, it is this API which enables the answering of questions such as ‘which sites were actively archived during the period…?’ or ‘how does frequency of capture vary across the archive, and over time?’.

**Fig. 3:**
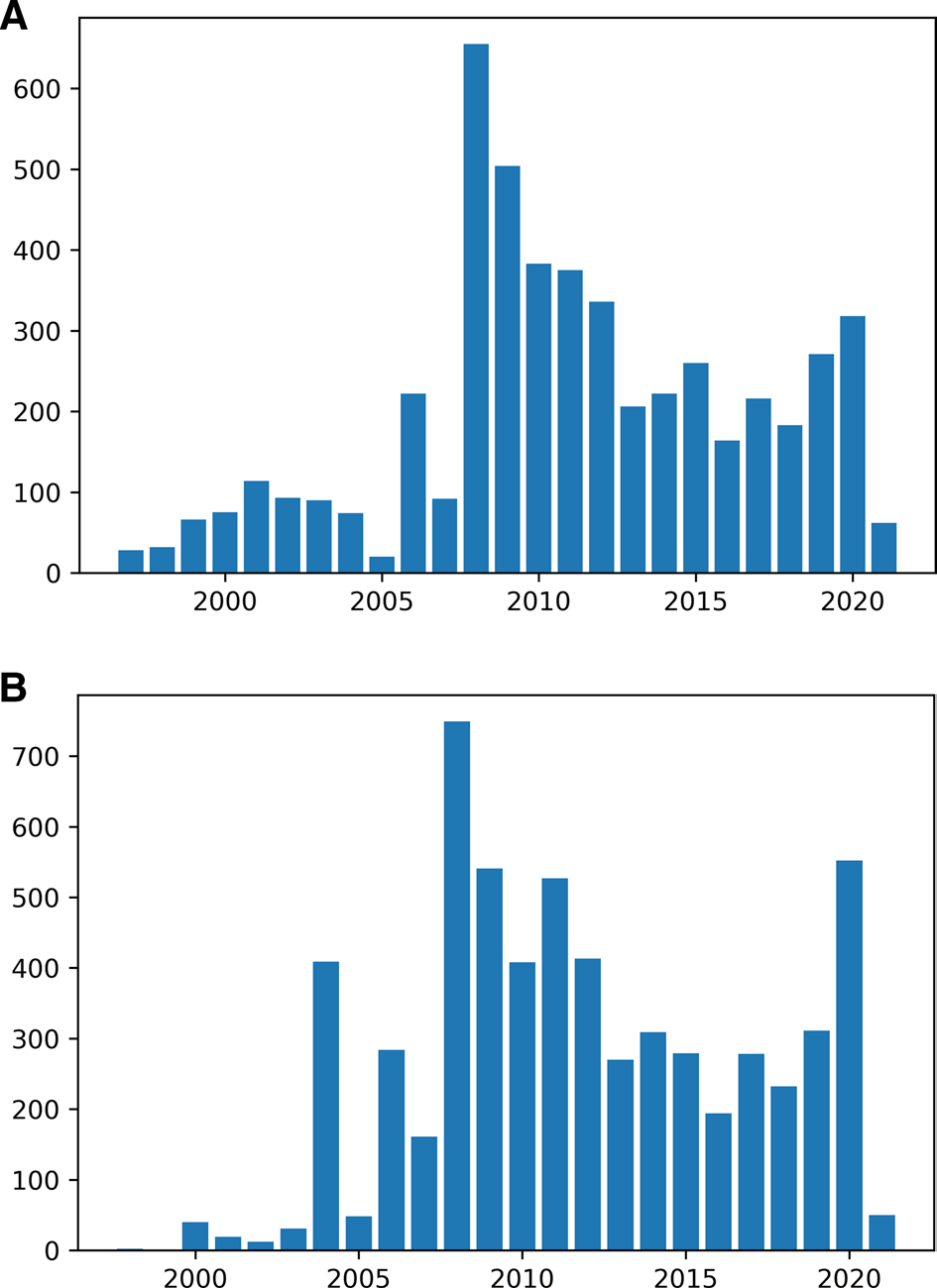
**a** Discovery entries by start date. **b** A–Z entries by first snapshot

The Notebook loads a file (to save time) of CDX data for all of the home pages (source: A–Z list) in the UKGWA and counts of pages by year of first snapshot (Fig. 3b). The equivalent summary of Discovery data, using the start date, is shown in Fig. 3a. While the charts are similar, there are some noticeable differences. The Discovery data have higher volumes pre-2005 because the catalogue date reflects the earliest document within a record, and some collections include web material which is held as a standard born-digital record and not in the web archive. The earliest snapshot of the Statistics Commission website is from January 2004 (The National Archives 2021i) and is catalogued under reference SF 1 (The National Archives 2021g). However, SF 2 (The National Archives 2021g) and SF 3 (The National Archives 2021h) contain documents published on the first and second websites of the Commission, the earliest from 2000. At the other end of the graph, there are no records for 2021 in the Discovery data. This highlights the fact that catalogues are manually maintained and entries are added according to workload and priorities—the web archive is competing with 1000 years of documents. The CDX data on the other hand are dynamically updated and reflect live archiving activities.

CDX can be used to analyse changes in a resource by combining other fields from the API. The ‘status’ is a standard internet response code used to identify whether the retrieval of a web resource has been successful, or not. Opening a website in a browser generally triggers a response code of 200, invisibly to the user, who will be more familiar with the jarring response of a 404 (Page not found) code. This code can identify failures in the archiving process, but is most useful for identifying redirections (301 or 302) which most commonly occur when migrating a site to a new domain. The CDX does not identify the location of redirection, but it is interesting when combined with the digest.

**Fig. 4:**
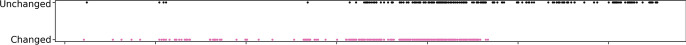
Frequency of changes in the Environment Agency’s homepage, 1996–2021

The digest (or checksum) is the output of a cryptographic hashing function which can be used to identify whether any changes have occurred from one snapshot to another. The key word here is ‘any’. Nothing in the checksum enables identification or quantification of change. Web documents are a combination of layout, navigational, informational (banners and popups), and content elements. Navigation menus and banners in particular are generally shared across all pages within a site. A change to the wording of the ‘Cookies on gov.uk’ pop-up message on www.gov.uk will reflect as a change in the checksum for every page on the domain even though nothing has fundamentally changed on those pages. The checksum in the case of a redirection is that of the redirection message sent to the browser not of the landing page, meaning a change in the redirected to page is not reflected in the checksum. This is illustrated by Fig. 4 for the home page of the Environment Agency (Environment Agency 2014).

The top row of black points indicates pages which are unchanged since the previous snapshot, while the bottom row (pink) indicates where changes have occurred. The plot shows how the frequency of capture increased from 2008 and the dense overlap of black and pink dots between 2008 and 2014 suggest an active page with frequent enough captures to record a high percentage of updates. After 2014, there are no further changes but the status code in the CDX data shows that the page was redirected from 4 May 2014 onwards (Environment Agency 2014). Despite the digest being a blunt tool for identifying change, it is useful for differentiating between a static and an active site.

**Fig. 5:**
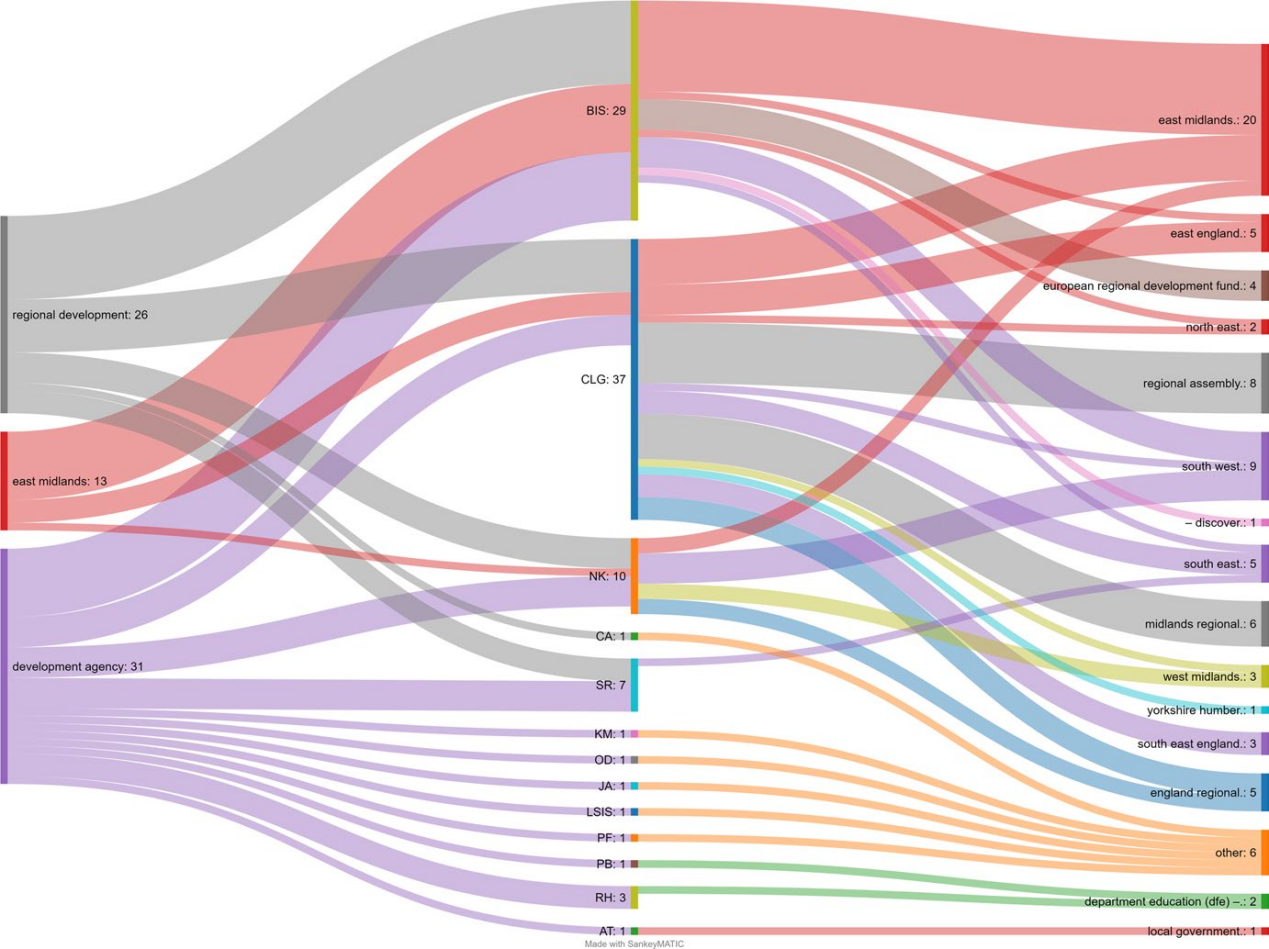
Sankey diagram showing Administrative History terms linked to A–Z terms via department

Notebook 1 ends with a visualisation (Sankey diagram) demonstrating linking data across the two catalogues, which is shown in Fig. 5. On the left are three administrative history phrases (development agency, regional development, East Midlands) which connect to department codes in the centre. The codes in turn connect to common phrases extracted from A–Z descriptions for websites from those departments. If there is no common phrase in a description, it is defaulted to ‘other’. The diagram is animated in a correctly rendered Notebook and provides an experimental view not possible without bringing together these two data sources.

The data used to produce these summaries are a mixture of metadata created for the purpose of filtering a catalogue (e.g. taxonomy codes), metadata generated by archival process (CDX), and unstructured text whose purpose is to provide information to a human reader (administrative histories and the A–Z index). In the case of the A–Z, there is some structure with many entries consisting of two parts separated by ‘–’, generally a department followed by a granular classification (e.g. GOV.UK Blog). Metadata extracted through an API is the ideal source for computational analysis, but in both cases there is scope to improve the APIs. There is no UKGWA taxonomy category, or other indicator, within Discovery, leading to using a keyword search to find UKGWA records. The CDX API has a lot of functionality (not discussed here), but URL is a mandatory parameter, so is unsuitable for speculative querying. Rather than having to scrape the data, the A–Z index could also be provided through an API.

In theory, Discovery could provide this index, but the URLs are not surfaced through the API, and a manually maintained spreadsheet was needed to provide the link between the two systems. Following the call by Moss et al. (2018) to ‘Reconfigure the Archive as Data to be Mined’ (Moss, Thomas, and Gollins 2018), work is in progress, though Project Omega (The National Archives 2021d), to re-engineer the catalogue as linked data which will improve the connections between the main archive and UKGWA. It will be worthwhile periodically revisiting these Notebooks as that project progresses.

This Notebook has introduced two ways to access archival catalogue information about the UKGWA: through the archive’s APIs, and scraping information from the web archive’s index. In doing so, the potential of bringing together multiple sources of data was demonstrated, but also the challenges of combining information which has not necessarily been designed with this purpose in mind.

### Notebook 2: Crawling the Government Web Archive

The first Notebook focused on available metadata and crawled the A–Z page of websites. To attempt any further analysis of the content or network structure of the UKGWA, crawling of websites is necessary. Python libraries designed for downloading and processing HTML files from the web are available, and in this case, the popular BeautifulSoup library has been implemented (Richardson 2020). The library includes functionality to programmatically open a web page (i.e. not in a browser), and extract text, hyperlinks, and other components of a web page. The Notebook is not a tutorial on web scraping but highlights some considerations when extracting information from UKGWA websites.

One feature which differentiates scraping a web archive from the live web is that links may point to other archived pages, or pages outside of the archive’s scope. The formats encountered during the creation of this Notebook are shown in Table 1.

**Table 1:** URL formats encountered in hyperlinks extracted from the UKGWA

URL type	Format	Example
Complete Address	Web archive prefix + collection + snapshot + full url	https://webarchive.nationalarchives.gov.uk/ukgwa/20141226000648/https://www.gov.uk/vehicle-tax
Snapshot Address	Snapshot + full url	20,141,226,000,648/https://www.gov.uk/vehicle-tax
Full url	Full url	https://www.gov.uk/vehicle-tax
Relative url	Page name	vehicle-tax or./vehicle-tax

Standardisation is a straightforward task, but it is important that the user is aware of these multiple formats. In the absence of a timestamp, it is necessary to inherit the timestamp of the page containing the link, and a relative link will be prefixed by the whole url of the parent page. Other links such as ‘mailto’ or shortcuts to sections (prefixed with a #) also appear, but they apply to web pages in general and are not specific to the web archive.

Table 1’s example is found in the 20,141,226,000,648 snapshot of www.gov.uk, but be aware that the snapshot referred to in the hyperlink does not necessarily exist. Following this link will redirect the user to a snapshot dated 20,141,226,073,441. This is only a seven-hour difference, but the discrepancy between link address and the archived copy can range from seconds to years. The http://www.environment-agency.gov.uk:80/who.html page was first captured 467 days later on Valentine’s Day 1998 (perhaps it felt unloved), but the hyperlink to it on the Environment Agency home page is labelled as 4 November 1996. Large discrepancies can be due to a difference in the crawling depth over time. In the early days of the web archive, often only the home page itself or the pages directly linked from it were crawled. Over time, crawling policies were changed to capture as much of a site’s content as possible. In this instance, it appears to be an oversight, or perhaps a technical hitch, as other links on the page were crawled. This was the first website captured by the UKGWA.

Two options are available to identify the true snapshot date: follow the link automatically resolving to the closest archived version; use the CDX API and apply one’s own logic to finding the desired snapshot date. Using built-in functionality in the archive is the easier option, but there may be performance considerations as following a URL involves retrieving that URL’s web content (possibly including images and videos). Many links will never be resolved because they point to sites outside of the scope of the archive. An NHS story about training toddlers to eat vegetables links to the BBC news page they sourced the story from (National Health Service 2014). This link has all the appearances of an archived page, including timestamp, but was not captured as it is outside the remit of UKGWA. This has a knock-on effect in network analysis where there is a mixture of bidirectional links, links confirmed as being one directional (both pages are archived), and links in one direction but lacking evidence that a return link does not exist (only one page is archived).

Content can be extracted from pages in order to perform text analysis, and the Notebook presents three methods for this task. The Python library used includes functions for extracting text, but navigation menus, and embedded javascript (code enabling dynamic functionality) is also included. The pages need to be pre-processed using boilerplate removal tools to strip out the menus (Kohlschütter et al. 2010). While these tools are effective, many home pages consist entirely of links and menus, resulting in an empty page following boilerplate removal. Applying the tool to the www.salt.gov.uk page provides an example of this occurring. While retained navigation menus can add unwanted noise when performing text analysis, they are important informational, and contextual, elements in their own right.

The Notebook concludes with a demonstration of Document Summarisation (Barrios et al. 2016) available through the gensim Python library (Řehůřek and Sojka 2010). This algorithm reduces the size of a document (by default to 20% of its original size) by extracting the sentences which are most representative of the whole text. In the demonstration, the hyperlinks from a selected home page are followed and the text of each page is read and summarised, following boilerplate removal. The summaries are collated and passed through the algorithm a second time, creating a c.100 word summary of the site. The summary is shown alongside the administrative history from Discovery (where available) for comparison.

The purpose of this demonstration is twofold. It shows the potential for computational methods to fill in the gaps of descriptive metadata when full text is available while highlighting the differences between an automated summary and an archivist curated description. Secondly, the Notebook environment is presented as a playground for experimentation and showcasing new technologies. The minimal setup required, the intuitive and low skill interface, and the availability of cutting edge technologies in open-source software libraries, mean it is easier than ever to build demonstrations like these which can be interacted with by technical and non-technical members of the archival community alike.

The Notebook reiterates the point about the archive as data. A lot of development effort is required to identify, extract, and re-format content, entities, and links from web pages in order to use them as data. This presents an insurmountable challenge to the majority of researchers, and even for an experienced developer adds extra effort and delay to the primary task of analysing the data. During the writing of this article, there were connectivity issues with the Discovery API from cloud platforms, as it was migrated to cloud infrastructure, and the output of the CDX API also underwent a major change, along with the web archive URL structure. Both of these issues required library code to be rewritten in order for the Notebooks to continue functioning. All of this raises the question of what archives can do to make their born-digital collections available as data, and how they can aid the sustainability of computational access methods, which will be discussed further in the next section.

## Discussion

The choice of using Notebooks to explore the UKGWA was made deliberately. At the beginning of 2020, TNA ran a Machine Learning Club (MLC) for staff from across the organisation. The aim was to demystify machine learning while giving hands-on experience of working with data and training models (Bell and Talboom 2022) through a series of workshops. Participants were from a range of backgrounds and only a few had programming skills, but the Notebook format allowed the organisers to present just enough code to understand the processes behind a machine learning workflow, accompanied by explanatory text and running instructions. The interactive nature of a Notebook meant participants could experiment with changing parameters, guided by instructions, or using different algorithms and seeing the effects their inputs had on results. As Candela (2020) points out similar benefits to using the Notebook environment: *‘Jupyter Notebooks lower many barriers to reproducibility by enabling scientists to combine code, results and text’* (Candela et al. 2020, 15). This idea is also echoed by the GLAM Workbench (Sherratt 2020) and the CLARIAH project (Melgar-Estrada et al. 2019).

One of the features of a Notebook, and a key driver of their popularity, is the ability to show the computer code (usually in the Python language) alongside the outputs (graphs, tables) that it produces. Early Notebooks were a blend of descriptive text and editable programming code possibly intimidating to a non-expert user, but Notebook widgets (drop down lists, buttons, structured output layouts) are an innovation which enables the creation of interactive Notebooks which are more intuitive to the average user, lowering the bar for entry to computational analysis. Dropdown lists are included in the Notebooks to cycle through examples, rather than having to edit any code. In order to demonstrate extraction and processing of data from the archive for the computationally minded user, some code is necessarily included to be read, not just executed. How much code to surface does depend on the technical sophistication of the audience. For a general audience complex, or lengthy, code which does not aid presentation is best hidden from view in library functions (for example, the code which extracts data from Discovery). These functions are not completely opaque as they are stored in the GitHub repository with the Notebook. A user who is interested in the possibilities of computational access can produce and explore the outputs in the Notebook, even experimenting with different parameters to see their effect, while someone with a programming background who wishes to understand how the API calls were made will find the relevant files in GitHub and may wish to reuse them for their own projects.

Following the experience of running machine learning workshops, Google Colab was selected as a platform for publishing these Notebooks. Recognising that not everyone has, or wants, a Google account, they can also be run on MyBinder, a cloud infrastructure using the Binder framework for hosting Jupyter Notebooks. With both platforms, the Notebooks run in a web browser, removing the need for local software installation. The experience for both developer and user is different for each environment. For the developer, Colab has a large array of Python software libraries pre-installed, whereas MyBinder must be instructed to install additional libraries. Colab also offers integration with Google Drive cloud storage enabling loading of user provided datasets, or exporting of results from the computation. This functionality is not available in a Binder setup. From the user perspective, Colab has a customised interface which includes support for markdown formatting, and the ability to collapse and run multiple sections together.

MyBinder can take longer to initiate the virtual machine it runs on, plus the extra installation time required to install libraries, although performance has improved during the writing of this article. It will also erase itself after 10 min of inactivity, while the Colab environment makes it easier to reconnect to a machine. Both environments are designed for experimentation, with the MyBinder documentation describing their infrastructure as being *‘…meant for interactive and ephemeral interactive coding, meaning that it is ideally suited for relatively short sessions’* (The Binder Team 2017), while Google acknowledges the constraints of running a freemium service: *‘In order to be able to offer computational resources for free, Colab needs to maintain the flexibility to adjust usage limits and hardware availability on the fly’* (Google 2021). Overall limits to how long a Notebook can remain open in Colab and MyBinder are 12 hours and 6 hours, respectively. There are also limits to the available processing power and working with large datasets or complex algorithms may go beyond the limits of free cloud infrastructure.

To set up a Notebook environment, a certain skill set is needed—some basic programming skills, and understanding of concepts and the infrastructure. This may not be available in every institution, but as GLAM Workbench demonstrates, it does not necessarily have to be the organisation themselves that sets up these Notebooks. It is a compromise between offering full computational access to users and having nothing available to them. As previously discussed, a balance should be struck between making them accessible to, and usable by, users without programming knowledge, while not oversimplifying to the extent that the computational aspects are completely hidden away from experienced users.

This exploration of the UKGWA would not have been possible without it operating under Crown Copyright (Open Government Licence) (The National Archives 2021k), making it a rare example of a web archive which is able to offer users access to the material without going into the reading room. Copyright issues mean that many archives are not able to offer direct access to their collection in this way, but that is not necessarily an insurmountable barrier. The HathiTrust offers a two-tier model for non-consumptive research (HathiTrust 2017). Firstly, they release extracts of words and named entities for basic content analysis. For more complex analysis, requiring the original text, researchers submit their own code or tools to run on HathiTrust servers, using their Data Capsules. Outside of the heritage sector, the Open Science Data Cloud offers a similar model. Scientific data have one common feature with web archives: scale. As the size of the archives becomes less practical to extract or download, it seems more likely that researchers will want to bring their tools to the data, rather than the other way around.

Even with the UKGWA operating under the Crown Copyright, there are still ethical issues to consider. As the UKGWA does not currently offer the capability to access content programmatically, web scraping was the only alternative for Notebook 2. Web Scraping is the process of using tools to automatically extract data from the Web to enable further analysis (Krotov and Silva 2018, 2). A number of guides have been released on this topic, Mitchell (2018) being the most comprehensive and updated version (Mitchell 2018). However, the ethical implications of scraping are only scarcely discussed. The social sciences, including criminology, have published a number of articles on web scraping as they understand the benefits of this field, but also worry about the ethical implications. The field of web scraping is legally ambiguous as ethical guidelines are not directly applicable to online research (Luscombe et al. 2021; Brewer et al. 2021).

As Krotov and Silva (2018) propose in their article, it would be best if organisations had ‘terms of use’ around web scraping, as currently the decision of scraping material that may be sensitive or copyrighted is left to the user, rather than having a preventable action in place (Krotov and Silva 2018). This is echoed by the other articles which are written from the researcher’s perspective and recommends them to consult ‘terms of use’ before considering web scraping (Mitchell 2018; Luscombe et al. 2021; Brewer et al. 2021). However, for most GLAM institutions providing online accessible digital material these guidelines are currently lacking.

A good example is the Internet Archive’s Responsible Crawling guidelines (Osborne 2018). Currently, the UKGWA does not have any guidelines such as this set up for the use of the web archive, TNA as a whole does have guidelines for web scrapers and bulk downloads, but prohibits anyone from using them without permission (The National Archives 2021l). As previously mentioned UKGWA is under Crown Copyright (Open Government Licence) and therefore available for research purposes (The National Archives 2021c). Although there are no specific guidelines for the UKGWA yet (possibly highlighting the novelty of computational access to the archive), it is important to remember that this is a live archive and crawling activity can impact daily archiving activities and potentially other users. Crawling therefore needs to be undertaken responsibly, and some basic guidelines could include:

- De-anonymise your activity by using a ‘User Agent’ which provides identifiable information including contact details when crawling, in case of problems.
- If possible restrict crawling to outside of working hours (8.00–18.00 in UK) to avoid impacting the service.
- Limit crawling rate to 3 URIs per second in working hours (if they are unavoidable), or 5 outside.
- Contact the web archiving team with any questions (see https://www.nationalarchives.gov.uk/webarchive/information/ for details).

This last point is important. Communicating with whomever is responsible for the archive about plans for web scraping is a common courtesy, and could open up alternative avenues for accessing the material. Being better informed about users’ scraping activities could influence future product development in terms of API features, scaling of computation resources, or creation of pre-processed data extracts (e.g. Named Entities). These guidelines could be adapted by any memory institution providing access to digital material that has the potential to be web scraped.

Web scraping in an ethical manner may not be suitable for a cloud hosted Notebook environment. At a speed of 5 URIs per second, crawling a meaningful subset of the archive could stretch the tenancy limits on a free cloud service. The over 300,000 snapshots of home pages would take over 1000 minutes to crawl at this rate. Our analysis of counting links from a sample of 200 pages found an average of 38 links from each page, with the number of links ranging from 1 to 243. At this rate, following links from one copy of each homepage would require 750 minutes of processing.

Generally, exponential growth in links is experienced as the depth of the crawl increases. Crawling is more suited to out of hours batch processing on a dedicated server, and using specialist tools. Institutions may consider creating downloadable datasets, such as the CDX extract accompanying Notebook 1. This can reduce traffic for common requests especially if there is API functionality available to enable updating with more recent data (as there is with CDX). As data volumes grow, a non-consumptive service becomes preferable, to be able to offer the best access for computational methods, but there will be large cost and resource implications for the institution. However, guidelines around web scraping are still strongly recommended as Mitchell (2018) highlights that the API may not be suitable for all purposes (Mitchell 2018).

Pages on UKGWA may be subject to takedowns (The National Archives 2021j)—removal from the open web—which while rare enough to have little impact on reproducibility of results, suggest care must be taken when scraping web content. There is a risk that taken down content could still be available in the community with archivists unaware what has been copied and researchers unaware they are holding closed content. The solution is to follow the GDPR principle of Storage Limitation (Information Commissioner’s Office 2021), having extracted entities or created an alternative data representation such as tf-idf (Ramos 2003).

Notebooks are a great way to explore access and to showcase the possibilities for researchers to go beyond keyword search. The Notebooks that were created serve three main purposes: to highlight the data sources available for computational analysis of the UKGWA; identify some features unique to the archived web a user must consider; encourage experimentation with this collection as a data source. Notebook 1 used four sources of data to connect the UKGWA to the wider Archive, three of which are publicly available, and one sourced internally. While this showed the possibilities available when data sources are brought together, it also showed that each of these data sources tells a different story about the collection. It highlighted that care needs to be taken when joining together data sources where even seemingly similar attributes such as record dates have subtly different meanings in the two systems. Notebook 2 went further in extracting links and content from the web archive, using the latter to generate automated summaries of web sites. It demonstrated that there is potential for interesting analysis but also points to the need for archives to do more to make their collections available as data to reduce the need for researchers to use crawling methods to acquire data and reinventing the pre-processing wheel.

Notebooks themselves may only be a temporary solution as organisations start to open their collections to computational access and explore its potential, but they can inform future development of access systems. By creating these Notebooks, a number of ethical issues were demonstrated that memory institutions should take into consideration. It may be that organisations themselves are not yet ready to offer this type of access, but as the web scraping possibilities show, it is something that terms of use should be set up for. Although these Notebooks are a first step to showcasing computational access for the UKGWA, having greater API functionality would expand their capabilities in a sustainable and ethical way. There is enthusiasm within TNA to make research datasets available to encourage engagement with the collection and to build a community who can inform the design of the next generation of computation ready interfaces to the web archive.

## Conclusion

This article explored the possibilities of using cloud hosted Notebooks to provide a different way into the UKGWA that goes beyond the current keyword search interface. The work builds upon other projects and examples using Notebooks and generous interfaces. It quickly became apparent that Notebooks are helpful to explore born-digital material using digital methods, within an environment that enables experimentation, is easy to share, and encourages reproducibility. The format allows a narrative explanation to be included alongside the code, enabling users to follow along at their own pace and in their own time. They are also a great way to advocate for computational access in the archival setting, as it shows what is possible when going beyond the popular keyword search.

There are some downsides to these kinds of Notebooks. By using free cloud infrastructure, they are reliant on continued funding for these services for sustainability, which is not guaranteed, and both services acknowledge their limitations. While Notebooks can provide alternative access methods to collections, they must be distinguished from managed services, such as a search system, which are built into the infrastructure of an organisation’s online service. As experimental products, they are not necessarily designed robustly, nor to be particularly efficient, and if they cease functioning (as the authors experienced), it is unlikely to be an institutional priority to provide dedicated support for them. The GLAM Workbench is an exception demonstrating that a dedicated individual can go a long way, with community support and good relationships with institutions. These factors are essential to the long-term sustainability of open-source projects.

Institutions cannot be expected to provide for all user needs, and Notebooks do seem to be a good in between for now. However, the main thing the authors would like to advocate is the importance for institutions to consider the ethical issues surrounding computational methods. Ideally, implementing an API, with guidelines, would be best, but this is not feasible for every institution and is not the only way to access this data in bulk. Before using web scraping as a data collection technique, it is also recommended to contact the institution in the first place to open more efficient alternatives. Nonetheless, it is important for institutions to think about the ethical implementations this may have for their own material and create helpful guidelines for users seeking computational access to this material.

## Data Availability

Data from UKGWA are openly accessible through their website. Notebooks created for this article are available on GitHub.

## Code availability

The code is available on GitHub: https://github.com/nationalarchives/UKGWA-computational-access. For a tutorial on how to use Notebooks and Binder, please access: https://reproducible-science-curriculum.github.io/sharing-RR-Jupyter. Further specifications regarding the accompanying Notebooks in this article can be found in the README file on the GitHub page.
